# Consolation in the aftermath of robberies resembles post-aggression consolation in chimpanzees

**DOI:** 10.1371/journal.pone.0177725

**Published:** 2017-05-31

**Authors:** Marie Rosenkrantz Lindegaard, Lasse Suonperä Liebst, Wim Bernasco, Marie Bruvik Heinskou, Richard Philpot, Mark Levine, Peter Verbeek

**Affiliations:** 1 Netherlands Institute for the Study of Crime and Law Enforcement (NSCR), Amsterdam, The Netherlands; 2 Department of Sociology, University of Copenhagen, Copenhagen, Denmark; 3 Department of Spatial Economics, Vrije Universiteit Amsterdam, Amsterdam, The Netherlands; 4 Department of Sociology and Social Work, Aalborg University, Aalborg, Denmark; 5 Department of Psychology, University of Exeter, Exeter, United Kingdom; 6 Department of Anthropology, University of Alabama at Birmingham, Birmingham, Alabama, United States of America; University of Medicine & Dentistry of NJ—New Jersey Medical School, UNITED STATES

## Abstract

Post-aggression consolation is assumed to occur in humans as well as in chimpanzees. While consolation following peer aggression has been observed in children, systematic evidence of consolation in human adults is rare. We used surveillance camera footage of the immediate aftermath of nonfatal robberies to observe the behaviors and characteristics of victims and bystanders. Consistent with empathy explanations, we found that consolation was linked to social closeness rather than physical closeness. While females were more likely to console than males, males and females were equally likely to be consoled. Furthermore, we show that high levels of threat during the robbery increased the likelihood of receiving consolation afterwards. These patterns resemble post-aggression consolation in chimpanzees and suggest that emotions of empathic concern are involved in consolation across humans and chimpanzees.

## Introduction

Within social philosophy, consolation is seen as a fundamental human achievement [1]. Consolation, in this view, soothes anxiety and our existential vulnerability, and draws on a shared sense of humanity, empathy, trust and perspective-taking. Scholarship on consolation dates back to the Greco-Roman world, and unlike mere empathic perspective-taking that does not necessitate action, consolation requires action in response to the recognition of the plight of the other [2].

While the extensive philosophical literature on consolation is extensive, few observational or ethological studies are available on human consolation in naturalistic settings. This paucity likely reflects the difficulty of carrying out unobtrusive and systematic observation of human behavior in natural conflict [3]. Here we introduce surveillance camera footage as a new method to systematically measure post-aggression consolation in adult humans. Specifically, we analyzed the immediate aftermath of nonfatal robberies to look for adult human consolation. Surveillance camera footage offers a unique and ethically sound way of studying adult behavior in high-danger contexts [4].

In nonhuman primates, consolation was first observed in chimpanzees (*Pan troglodytes*). Consolation was termed as such because it visibly reduced distress in victims of aggression [5, 6] and subsequent research has confirmed this function [6, 7]. Behaviorally, consolation in nonhuman primates is commonly defined as interactions in which an uninvolved bystander initiates friendly contact with a recent recipient of aggression [8]. Consolation so defined is a highly replicable finding in chimpanzee populations [7, 9, 10], and has since been further documented in bonobos (*Pan paniscus*) [11, 12], mountain gorillas (*Gorilla beringei beringei*) [13], Western lowland gorillas (*Gorilla gorilla gorilla*) [14], and Tonkean macaques (*Macaca tonkeana*) [15]. In monkeys, bystanders have also been observed to offer friendly contact when solicited to do so by victims (e.g., *Cebus apella*) [16, 17]. As later research showed, the differentiation between (a) consolation initiated by the bystander (“consolation proper”), and (b) friendly bystander contact solicited by recipients of aggression, is important to understand the functional significance as well as the cognitive and emotional implications of the behavior [18]. Indicative of convergent evolution, consolation, as defined for nonhuman primates, has now also been observed in non-primate species such as canids, corvids, rodents and elephants [19].

Research on post-aggression consolation in humans has mainly focused on children from various cultures [20–22]. For example, Japanese children as young as 4 years of age offered consolation to victims of peer aggression. The frequency of this consolation increased rapidly between 4 and 5 years of age, most likely a reflection of developing socio-cognitive skills such as the ability to attribute mental states to others (“Theory of Mind”), and to understand that another’s point of view about an issue may contrast with one’s own [23, 24]. In this context, consolation in human adults can be seen as an empathy-related behavior that involves sharing another’s distress via emotional contagion, combined with sympathizing and perspective-taking [25–27].

Almost three decades ago, in 1989, de Waal called for more naturalistic studies on human adults so as to overcome the view that the human-nonhuman primate comparisons simply show that “other primates are *mentally* like human children” [28]. Here we compare post-aggression consolation in human adults with post-aggression consolation that prior research has identified in adult chimpanzees. Through this comparison with our evolutionary closest kin, we shed light on the mental processes involved in post-aggression consolation across human and non-human primates [29]. Such comparison is in agreement with the principle of evolutionary parsimony advocated by de Waal, suggesting that if closely related species behave similarly, then the underlying mental processes are presumably similar too [29].

A growing body of research across chimpanzee populations in varied social conditions shows how bystander affiliation may serve vital *peacemaking* and *peacekeeping* functions. For example, chimpanzee bystander affiliation can appease aggressors—especially if the bystander has a close relationship with the aggressor [8]—which can protect bystanders against redirected aggression [30]. Chimpanzees also offer consolation to victims of aggression, often by hugging and kissing the victim. It can be assumed that these different types of bystander affiliation and the functions that they serve rely on different social and individual motivations [31].

Post-aggression consolation in chimpanzees is promoted by social closeness [9, 32, 33]. Although several earlier studies did not find evidence associating age and sex with the likelihood of providing consolation [6, 34, 35], a recent study that draws on data from an exceptionally large sample of spontaneous consolation in chimpanzees, offers compelling evidence that female individuals are more likely to provide post-aggression consolation than males [9]. Given that social closeness is known to facilitate empathy in both humans and other animals and that females are generally more empathic than males, these findings are consistent with an empathy explanation [36].

We analyzed the video-recorded behavior of 249 individuals who were present in the aftermath of 22 commercial robberies, immediately after the offenders had left the scene. We investigated whether and how *providing* and *receiving* consolation were affected by social closeness and gender. Specifically, as documented in chimpanzees and predicted by the empathy explanation, we hypothesized that consolation is promoted by social closeness and that females are more likely than males to provide consolation.

According to theory, and supported by empirical findings in ethology and in the social sciences, we hypothesized that consolation might be further facilitated by additional factors. First, based on the homophily principle that similarity breeds connection [37], we conjectured that consolation is more likely between individuals that are similar in terms of ethnicity and age; two of the factors that create the strongest divides in our personal environments [37]. This hypothesis is supported by findings demonstrating that empathic response is amplified by similarity and that individuals are more likely to help distressed others that are similar to themselves in terms of age and ethnicity [38–40].

Second, a comprehensive literature in social psychology has studied the conditions under which human bystanders respond to the needs of victims in emergency situations. A recurring finding is the 'bystander effect', the phenomenon that individual bystanders are less likely to intervene with increasing numbers of other bystanders present [41, 42]. The bystander effect is typically ascribed to a psychological process by which the presence of other bystanders diffuses the responsibility for helping. To test for the bystander effect, we measured the total numbers of individuals present in the aftermath of the robbery.

Because individuals who have better opportunities to respond to distressed victims are reportedly more likely to respond [42], and because physical proximity between individuals has been demonstrated to facilitate consolation among human children [23], we measured the physical distance between all pairs of individuals at the start of the robbery aftermath. We hypothesized that consolation is more likely to occur when individuals are in closer proximity to each other.

A third theoretical framework from which we draw is the aversive-arousal reduction hypothesis [43]. It proposes that the empathic arousal evoked by witnessing the distress of another person is an unpleasant feeling, which can be alleviated by victim helping. As human bystander research suggests that people are more likely to help distressed victims in high-danger compared to low-danger situations [42], we also measured the degree of threat that potential recipients of consolation had been exposed to during the robbery. We hypothesized that a higher level of threat exposure would make an individual more likely to receive consolation from others. In addition, but more tentatively, we hypothesized that arousal itself, independent of its origin, is reduced by providing consolation. To examine this possibility, we also included the degree of threat that potential providers of consolation had been exposed to during the robbery. This allowed us to test whether the individuals most severely affected by threat during the robbery would be more likely to console others than individuals less affected.

Finally, sociological theory and cross-cultural empirical studies on gender suggest that social norms depict females to be more vulnerable, and hence more eligible for helping behavior than males [44]—particularly in dangerous situations [45]. It has also been demonstrated that it is more socially acceptable to touch women than men [46]. To investigate the validity of these findings–which point towards females as more likely recipients of consolation than males—we included the gender of potential consolation recipients as a variable of interest.

Based on the premise that bystanders are in a state of lower emotional involvement than victims, all prior empirical studies on post-aggression consolation have assumed that victims and bystanders are discrete categories. This implies that only bystanders and not victims of violence can provide consolation, and that only victims and not bystanders can receive it. However, distinctions between victims and bystanders in robberies can be fluid, and emotional involvement need not necessarily differentiate strongly between those who have been exposed to aggression firsthand (victims) and those who have been exposed by proxy (bystanders). For this reason, we did not label the individuals as either victims or bystanders, but instead measured for each individual the extent to which they had been subjected to threat during the preceding robbery. The observed level of threat directed towards the individual was thus included as a variable. To reiterate, this was not used to create a dichotomy between victims and bystanders.

In addition, we did not make any assumptions on who could console whom. We considered all dyads in both directions as potential consolation opportunities. This approach made it possible to analyze multiple instances of consolation between multiple individuals involved in the same incident. Simultaneously, this allowed the observation of unexpected behavioral patterns. For example, it allowed us to include in the analysis potential cases in which an individual who experienced a brutal attack could console an individual who only witnessed the event. Further details of the analytical approach are provided in the ‘Materials and methods’ section.

Consolation was defined as affiliative physical contact, including not only ‘hugging’, but also gentle touching of the target’s head, upper body, arms or hands (see S1–S3 Tables for the codesheets that were used).

## Materials and methods

### CCTV footage files

The Police departments of Amsterdam and Rotterdam (the Netherlands) gave the first and third author access to unprocessed CCTV footage of incidents that the police had classified as a robbery or as an attempted robbery. A robbery was defined as an event where goods or money are taken by force or by threat of force. Access was provided under the condition that data would be securely stored, not be publicly shared, and that the identity of the individuals visible in the footage would be protected. The research was authorized by the Dutch Ministry of Justice and approved by the Ethics Committee for Legal and Criminological Research (CERCO) of the Faculty of Law of Vrije Universiteit Amsterdam.

In all cases, the footage had been recorded by CCTV systems installed by the proprietors of the retail businesses in which the robberies took place. The materials had been stored on police computers for investigative purposes. Footage was not removed when the suspects were prosecuted or convicted. The police department of Rotterdam provided access to all robbery footage collected between June 2014 and August 2015. The police department of Amsterdam provided all footage that had been used in producing reality TV broadcastings aimed at eliciting public information. The material obtained for the research was all original, unedited material.

The video files were stored in many different formats; often these were proprietary formats that could only be watched with a designated video player program. Therefore, before coding commenced, the program CamStudio was used to convert all video files to AVI format. Occasionally, Windows Movie Maker was used to amalgamate footage from multiple cameras on a single site together into one file. Cases where images were blurred or where coverage of the interactions among individuals involved was incomplete were excluded from analysis. Of the 58 remaining cases, 32 did not include footage of the aftermath of the robbery and were thus excluded. Four of the remaining 26 cases contained less than 2 seconds of aftermath footage. These four cases were also not used in the analysis, as we judged this time interval too short for anyone to be able to provide consolation. The final sample for analysis comprised of 22 cases, the minimum footage duration was 14 seconds.

### Coding scheme and observer instructions

Using the Observer XT software [47], all footage—both the robbery and its aftermath—was observed and independently coded by two observers. Prior to coding, these two observers undertook two days of Observer XT training; with particular emphasis given to the tailored coding scheme that included measures of setting, individual and dyad characteristics.

As summarized in S1 Table in supplementary materials, for each setting analyzed we report (1) the number of individuals present in the aftermath, (2) the function of the location, e.g., supermarket or hotel lobby, (3) the size of the setting, coded as ‘large’ if it would take an average person more than five seconds to walk from one side to the other, or ‘small’ otherwise.

Robbery victimization was defined as the psychological or physical suffering induced by the presence or activities of offenders, and its extent was assumed to vary between individuals. The first recorded individual characteristic of interest was (1) the level of victimization the individual had suffered during the preceding robbery. This was either (1a) stood close to the offender, (1b) received a threat only, (1c) suffered physical force, (1d), suffered both threat and physical force, or (1e) none of these (for details see S2 Table in supplementary materials). Other observed individual characteristics included (2) whether the individual was an employee or a customer (based on clothing, the type of activity the person displayed, or on position in the room, e.g., behind a desk), (3) whether the individual was male or female (based on body form, hair, clothing and other body wear), (4) the estimated age of the individual, (5) the ethnic origin of the individual based on hair and skin color.

As summarized in S3 Table in the supplementary materials, observed dyad characteristics included (1) a light form of consolation from provider towards recipient, whereby the provider touched a hand or lower or upper arm of the recipient, (2) a strong form of consolation from provider towards recipient, whereby the provider initiated and completed upper body contact including hugging and embracing of the recipient. The distinction between ‘light’ and ‘strong’ consolation was not based on prior literature, but on initial qualitative exploration of the material. As it has been demonstrated that the perceived intimacy/intrusiveness of being physically touched by others depends to a large extent on which area of the body is being touched [46], it seemed useful to make the distinction between light and strong consolation dependent on the area of the body that was touched. The duration of consolation was not taken into account. As the footage did not have recorded sound, words of consolation and other verbal communication between the subjects could not be recorded. We also measured (3) the physical distance between two individuals at the start of the robbery aftermath, coded as ‘large’ if they were located more than 2 meters apart, and ‘small’ otherwise.

The two observers–who worked in isolation—coded the characteristics and behavior of every individual present in the footage of each of the robberies. Consolation behavior was coded directionally, identifying both the provider and the recipient of consolation.

### Assessment of coding reliability

To analyze the amount of correspondence between the measures for both observers, each individual visible in the aftermath of a robbery was assigned a unique reference number. To that aim, and before observations and coding commenced, for each robbery a numbered list was created describing each individual by gender, hair style and hair color, size, clothing and position at the beginning of the robbery aftermath. Without such a detailed list, coders can easily mix up individuals [48].

Four measures were central to the analyses. To inspect levels of agreement on these four coded measures, we calculated the Krippendorff’s alpha (Kα) coefficient of interrater reliability between coders [49]. This is a generic measure of the level of correspondence between observers, whereby values at or below 0 indicate no or negative correspondence, and values near the maximum of 1 imply perfect agreement. The results were (1) Kα = .81 for the level of victimization during preceding robbery, (2) Kα = .99 for whether an individual was an employee or a customer, (3) Kα = .79 for physical distance, and Kα = .78 for (light or strong) consolation. As it is customary to consider measures reliable when α ≥ .80 [49], these values are acceptable. With all four measures included in the same multivariate regression analysis, it is particularly reassuring that not a single measure is far below .80.

As discussed above, the distinction between light consolation (hand and upper arm contact) and strong consolation (upper body contact) was not unambiguous. The Krippendorff α was only .54 and .67 for light and strong consolation, respectively. Follwowing recommendations in the methodological literature on interrater reliability [49] we combined the light and strong consolation measures into a single consolation measure. Intercoder reliability on this combined measure was acceptable (Kα = .78).

Given that the observers did not perfectly agree on all measures (as Kα < 1) we had to decide on which codes to use in the analyses. This was done with a simple randomization procedure: We selected all observations of one of the two coders by throwing a dice.

### Descriptive statistics and data file setup

The coded materials can be explored at three separate levels of analysis, namely (1) incident level (Ni = 22), in which in each robbery is a single observation, (2) individual level (N_s_ = 249), where the individual subjects visible in the post-robbery footage are the units of analysis, and (3) dyad level (N_d_ = 3680), where all pairs of individuals present in the aftermath of the same robbery are considered. Each dyad represents a potential directional consolation relationship, and thus the dyads A→B and B→A are different; the first one indicating individual A providing consolation to B, and the second one indicating individual B providing consolation to A. This implies that bystanders can console victims as well as other bystanders, and that the same holds for victims.

S1 File is the single source data file with directed dyads as the unit of analysis. S2 File is the Stata script that can be used to replicate all descriptive statistics reported below as well as the model estimates described in the main text and in the supplementary information. The variable ROBB_ID identifies unique incidents. Within each incident, variables PROV_ID and RECI_ID identify unique individuals, such that PROV_ID identifies a potential provider of consolation and RECI_ID identifies a potential receiver of consolation. As self-consolation (in the form of self-touching) is a related phenomenon but distinct from consolation [50], this behavior was excluded from the analyses, so that PROV_ID ≠ RECI_ID for all records. Other variables included are N_PEOPLE to indicate the number of people (victims and bystanders) present, PROV_FEM and RECI_FEM to denote the gender of potential consolation providers and receivers, PROV_ROLE and RECI_ROLE to denote their role (employee or customer), and PROV_AGE and RECI_AGE and PROV_ETNIC and RECI_ETNIC to code their estimated ages and ethnicities. PROV_VICTIM indicates whether a potential provider of consolation was subjected to weapon threat or physical force in the robbery incident. RECI_VICTIM indicates whether a potential recipient of consolation was subjected to weapon threat or physical force in the robbery incident.

DISTANCE indicates the physical distance between the potential provider and the potential recipient of consolation. The binary response variable CONSOLATION indicates whether or not the potential provider (PROV_ID) consoled the potential recipient (RECI_ID).

At the *incident level*, there are 22 robbery aftermath recordings. The aftermath starts the moment the last offender leaves the scene and terminates when the recording (as obtained from the police files) is cut off or completed. The mean duration of the 22 recordings is 94 seconds (range is 14–324 seconds). Nine recordings are less than a minute, seven last longer than two minutes. Robberies took place in various settings, the most common type (n = 8) being a supermarket. The mean number of individuals present in the aftermath of the robberies is 11; the median is 10 (range is 3–27). In most cases, the individuals present included males and females. There was only one all-male case and only one all-female case. As the events are commercial robberies, the individuals present in the cases are typically either employees (including proprietors) or customers. The mean number of customers was 7.5 and the mean number of employees was 3.6. If we call ‘victims’ those who had experienced a threat with a weapon or who had been subjected to physical force during the preceding robbery, and ‘bystanders’ those who had not, the number of victims ranges between 1 and 4 (mean is 2), the number of bystanders ranges between 0 and 26 (mean is 9).

At the *individual level*, the footage included 249 individuals, 132 males and 117 females, 166 customers, 79 employees and 4 individuals that could not be identified as either employees or customers. Threat level during the preceding robbery was categorized as “no threat” (190 individuals), “stand near offender” (15), “threat with weapon” (23), “physical force” (5), “weapon threat and physical force” (14) and “unknown” (2). Thus, if a victim is defined as an individual who has received a threat and/or has been subjected to physical force during the robbery, and if a bystander is defined as an individual who was present but not a victim, the 249 individuals comprise of 42 victims, 205 bystanders and 2 individuals whom could not be assigned to either category. Ethnicity was categorized as White (162 individuals), Moroccan or Turkish (41), Black (26), Hindustan (5), Asian (2), other ethnicity (7) and unknown (6 individuals). Age was estimated in years, and observers were allowed to code “Unknown”. Aggregated to 10-year categories, the distribution was 11–20 (49 individuals), 21–30 (104), 31–40 (24), 51–60 (16), 61+ (7) and unknown (5 individuals).

At the dyadic level there were 3710 pairwise combinations. The gender distribution was 1188 (32.0%) male-male, 784 (21.1%) male-female and 1738 (46.8%) male-female. Of the 3658 dyads where the roles of both individuals were known, 232 (6.3%) were employee-employee. Only 96 (2.6%) of the dyads were located within 2 meters from each other at the start of the aftermath of the robbery. Victim-victim dyads (58) accounted for 1.6% of the dyads, victim-bystander dyads (796) accounted for 21.5%, and the majority (2856, 77.0%) were bystander-bystander dyads. Cases containing variables with unknown values were excluded from the analyses on a case-by-case basis. For example, individuals with unknown ethnicity were only excluded if ethnicity was part of the analysis.

### Statistical model

The standard methodology used in previous research on consolation is the PC-MC method [5, 6]. This method compares observed post-conflict (PC) interactions between victims and bystanders with matched controls (MC), i.e., observed interactions between the same individuals in situations not preceded by conflict. Our material did not allow this method, as we have no access to observations of interactions between the individuals prior to their interaction in the post-robbery CCTV footage. Instead, we adopted a regression approach that models the likelihood of post-robbery consolation as a function of specific characteristics of the dyads (e.g., whether the potential provider is female, and the distance between them).

A strategy that maximally uses the available data is to define the individuals present in the aftermath of a robbery incident as a social network. This allows examination of every possible relation between two individuals in a network as a potential opportunity for consolation. Whether or not consolation is provided is likely to depend on the characteristics of the potential provider, on the characteristics of the potential receiver, on the characteristics of the setting, and on the social and physical distance between potential provider and receiver.

Gravity models [51, 52] are well equipped to handle this type of data. They have been applied predominantly in economic geography for modeling flows of people, goods and information between spatial units of analysis, but have also been used in other disciplines, such as anthropology [53], to model the spatial organization of human activity. Moreover, gravity models are equally applicable to the analysis of dyadic relations in non-spatial network data [54]. In the application of a gravity model to consolation, we consider all possible N × (N-1) pairs of individuals present in the aftermath of a robbery, where N is the number of individuals. For example, in a robbery aftermath with three persons A, B and C, there are 6 consolation possibilities, namely A→B, B→A, A→C, C→A, B→C and C→B. Gravity models predict which factors determine whether an individual X provides consolation to individual Y, conditional on the characteristics of X, the characteristics of Y, the characteristics of the setting, and the social distance between X and Y.

Note that in the gravity model the observations are potential consolation relations. Only a small minority of the potential is realized and leads to consolation. We use the terms ‘provider’ and ‘recipient’ throughout, to prevent the cumbersome notation of ‘potential consolation provider’ and ‘potential consolation recipient’. As consolation is either provided or not, we used a logit regression equation:

$$\text{log}\left(\frac{p_{ij}}{1-p_{ij}}\right)=\beta_{1}X_{1ij}+\beta_{2}X_{2ij}+\ldots +\beta_{k}X_{kij}$$

Where *p*_*ij*_ is the probability that individual *i* provides consolation to individual *j*, where *X*_*kij*_ are the characteristics of the robbery event, individual *i*, individual *j*, or their relation to each other, and *β*_*k*_ are regression weights. The logit command of software package Stata/MP version 11.2 was used to estimate the models.

The structure of the dyadic data prohibits a straightforward maximum likelihood estimation of the logit model. The maximum likelihood logit model assumes independent observations, but dyads challenge this assumption for two reasons. First, dyads are nested in robberies, and may thus share unobserved characteristics with other dyads in the same robbery. Second, within a given robbery incident the dyads together represent a network structure. As a result, dyads involving the same individual as a provider of consolation or as a receiver of consolation, share unobserved characteristics of that individual. Both types of dependencies generate downwardly biased standard errors of maximum likelihood estimates. To account for these dependencies, we used the multiple regression quadratic assignment procedure (MRQAP) to estimate the logit models. The MRQAP is a statistical strategy to test regression coefficient in situations where independence assumptions are violated, and is typically used to correct for interdependencies in dyadic network data [54, 55]. We used the Stata module ‘qap’ to implement the quadratic assignment procedure.

### Diagnostics

We checked for linear relations amongst the explanatory variables for all models by calculating variance inflation factors of the explanatory variables and the condition numbers of the matrices of independent variables [56]. The results are satisfactory and are not indicative of degrading collinearity issues. All variance inflation factors in all estimated models were below 1.25, where values above 10 are generally seen as potentially problematic. The condition numbers of all models were below 5, whereas values above 30 indicate potentially serious collinearity [56].

## Results

The binary response variable in our analysis is whether consolation is provided by one individual to another. This variable is observed for every pairwise combination of individuals in the aftermath of the same robbery. This setup allows for the observation of a variety of patterns, including a single individual providing consolation to multiple others, and a single individual being consoled by multiple others. Consolation was *not* observed in the aftermath of 4 (18.2%) of the 22 robberies. In the remaining 18 cases, at least one person consoled at least one other person. Of the 249 individuals that were observed in the aftermath of the 22 robberies, 38 (15.3%) provided consolation to others, and 24 (9.6%) received consolation from others.

As our interest in consolation behavior focused on who is providing consolation to whom, we analyzed the data at the dyadic level. For each robbery *i* we considered all possible *N*_*i*_ *× (N*_*i*_*-1)* (directed) pairs of individuals present in its aftermath, where *N*_*i*_ is the number of individuals present in the aftermath of robbery *i*. The 249 individuals in 22 robberies included 3710 dyads. Using a logit model, we assessed which factors made consolation less or more likely to take place between the two members of a pair. Table 1 displays the roles of the four most significant factors.

**Table 1:** Logit analysis of consolation among 3680 dyads^4^. Quadratic assignment procedure.

Explanatory variable	b^1^	or^2^	p^3^
Socially close	1.999	7.38	.000
Potential provider female	.978	2.66	.002
Potential recipient is victim	3.593	36.34	.000
Number of subjects in aftermath	.019	1.02	.000

^1^ estimate (average of 1000 iterations)

^2^ odds ratio = exp(b)

^3^ p-value (based on 1000 iterations)

^4^ Four individuals’ roles (employee or customer) were unknown, affecting 30 dyads in total.

Social closeness predicted consolation. When both potential provider and potential recipient were employees of the same business (and thus presumably familiar having shared common experiences), the odds of consolation were 7.4 times greater than when either one or both of the dyad, were customers. Females were more likely to provide consolation than males. The odds of consolation were 2.7 times greater when the potential provider was female. Although some males provided consolation, females were more likely to offer it. Consolation was more likely to be provided to individuals exposed to high levels of threat (i.e., exposure to physical force or weapon threat). Victimization of a prospective recipient during the preceding robbery was the most important factor predicting consolation. For victims of either force or threat with a weapon, the odds of receiving consolation increased by a factor of 36.3.

To account for an unresponsive bystander effect due to a diffusion of responsibility [41], the model included the total number of individuals present in the robbery aftermath. Contrary to the hypothesized bystander effect, the odds of being consoled increased by two percent for every additional individual present in the aftermath of the robbery. To assess whether consolation is facilitated by opportunity, a model was estimated that included the physical distance between dyad members, measured by whether the pair were more than 2 meters apart at the start of the robbery aftermath. The results, reported in S4 Table, demonstrate that physical distance has no significant effect on the likelihood of providing consolation.

Although it has been argued that bystanders tend to help others in order to reduce their own stress [57], the results presented in S5 Table demonstrate no significant relationship between exposure to weapon threat or physical force and the likeliness of *providing* consolation. High levels of threat entitled individuals to receive consolation but did not make them more likely to provide it. Moreover, we did not find a relationship between *receiving* consolation and gender (see S6 Table). Exposure to weapon threat or physical force, not gender, determined whether individuals were eligible to receive consolation. Male victims were as likely as female victims to be touched by female consolers.

Based on the homophily principle, we tested whether consolation was more likely between individuals similar in terms of age and ethnicity. Individuals were coded as similar in terms of age if their estimated ages differed by less than 10 years (dyads with missing age estimates were removed from the analysis). Individuals were coded as ethnically similar if they were estimated to belong to the same ethnic group (dyads with missing ethnicity estimates were removed from the analysis and individuals in the ‘other’ category were never coded as ethnically similar to anyone). The outcomes of these models are displayed in S7 Table, and show that similarity based on age or ethnicity was unrelated to the probability of consolation.

Finally, all potentially relevant factors measured were included simultaneously in a single model. The results are displayed in Table 2, and again confirm that consolation is significantly more likely to be provided amongst affiliated individuals (i.e., employees), to be given by females, to be received by victims of threat and force (rather than received by bystanders) and to be provided when more individuals are present. All other potential factors were non-significant.

**Table 2:** Logit analysis of consolation among 3364 dyads^4^. Quadratic assignment procedure.

Explanatory variable	b^1^	or^2^	p^3^
Socially close	2.040	7.69	.000
Potential provider female	1.002	2.72	.000
Potential recipient is victim	3.500	33.12	.000
Number of subjects in aftermath	.037	1.04	.000
Physically close (< 2m)	.991	2.69	n.s.
Same age group	.176	1.19	n.s.
Same ethnic group	.032	1.03	n.s.
Potential provider is victim	-.884	0.41	n.s.
Potential recipient is female	.439	1.55	n.s.

^1^ estimate (average of 1000 iterations)

^2^ odds ratio = exp(b)

^3^ p-value (based on 1000 iterations), n.s. = p ≥ .05

^4^ Missing values on 346 dyads due to unknown values on the explanatory variables

## Discussion

Contemporary views in primatology consider empathic perspective-taking: putting oneself *emotionally and cognitively* into the other’s shoes, to be an integral component of human and nonhuman primate consolation [58]. According to this view, a chimpanzee bystander who offers consolation most likely acts on empathic perspective-taking, while a monkey bystander that offers comfort after being solicited to do so by a victim does not.

In the present study we showed that among human adults, as has been found in studies with chimpanzees, social closeness increased the likelihood of post-aggression consolation. Our study thus provides additional evidence that pro-social behavior is more likely among people who are familiar [27]. It might be the case that it is easier for familiars to interpret each other’s signals of distress [59]. Consolation may also be a way of establishing relationships or strengthening existing ones [60]. Note that unlike measures of social closeness in other studies, which are usually based on observed behavior that could indicate close affiliation (e.g., grooming, food-sharing), our measure of social closeness was based on the assumption that employees know each other and share similar experiences. This assumption does not typically hold for customers, nor for employees and customers.

In line with recent evidence on consolation in chimpanzees [9], we also found that females were more likely to console than males. This supports the notion that females are more prone to empathy-driven pro-social behavior than males [61–64]. One explanation for this finding is that females may be more sensitive to the emotional signals of others [65] due to maternal functions, social upbringing [66, 67] and cultural expectations [68]. Across cultures, it is also more acceptable for females than males to communicate through touching [46]. Taken together, the above results are consistent with an empathy-based explanation of differences in consolation, and suggest homologous mental processes of empathy in humans and chimpanzees.

Interestingly, females were not more likely to receive consolation than males. This challenges previous findings suggesting differential gender roles, particularly in dangerous situations [44, 45]. It also challenges studies showing that it is more socially acceptable to touch women than men [46]. Furthermore, our findings showed that for a given pair of individuals, the likelihood of consolation increased with the number of other individuals present. This contradicts the hypothesized bystander effect and confirms recent evidence that violence may diminish the bystander effect [42] or even reverse it [4]. Unlike prior findings on consolation in bonobos [12], our findings demonstrate that the physical distance between two individuals does not affect the probability of consolation. This suggests that providing consolation depends more on motivation than on opportunity.

Subjects who had been confronted with physical force or threats with weapons were much more likely to receive consolation than subjects who had experienced lower levels of threat. This shows the importance of considering threat levels while studying consolation, potentially also in studies of non-human apes.

The finding that individuals who had been subjected to physical violence or threat of violence during the robbery were not more likely to provide consolation to others, suggests that consolation was *not* primarily provided as a means of reducing one’s own stress; as the aversive-arousal reduction hypothesis would predict. Rather consolation was provided as a means of reducing the stress of others, thus supporting the empathy hypothesis. Future studies need to investigate whether consolation in adults also exists in low intensity conflict, and whether it follows the same patterns found here in high intensity conflicts.

Among humans, a sense of shared humanity is believed to mediate consolation [2]. Does a shared sense of ‘chimpness’ mediate consolation in adult chimpanzees? At the perceptual level, it appears that chimpanzees share such a common chimpness. Chimpanzees demonstrate *self-recognition* by passing the mirror self-recognition test [69] and exhibit *self-awareness* by distinguishing in real time an object that is under their control from one that is not [70]. Chimpanzees discriminate chimpanzee faces from the faces of other primate species, recognize kin relations by looking at conspecific faces, and tell apart and categorize expressions that are modelled by the faces of other chimpanzees [71]. Fights arouse special attention in chimpanzees. Controlled video tests of emotional scenes show that chimpanzees pay more attention to conspecific aggressive scenes than to other social situations, including play and general excitement [72].

Comparative research across species can offer clues about how species-typical social experiences and perception may be a factor in consolation. Delineating the roots and components of consolation through comparative research reminds us that the differences in the mental lives of animals are one of degree and not of kind. This ought to have implications for the practices of behavioral sciences and for the way we attend to animals other than humans.

## Supporting information

S1 File ConsolationRobbery.csv (datafile). (CSV)

S2 File ConsolationRobberyStataCode.do (Stata script analysis). (DO)

S1 Table Codesheet for static variables during aftermath of robbery. (DOCX)

S2 Table Codesheet of dynamic variables during robbery. Partial ethogram of offender behavior.
(DOCX)

S3 Table Codesheet for dynamic variables during aftermath of robbery (ethogram). (DOCX)

S4 Table Logit analysis of consolation among 3680 dyads. Quadratic assignment procedure.
(DOCX)

S5 Table Logit analysis of consolation among 3650 dyads. Quadratic assignment procedure.
(DOCX)

S6 Table Logit analysis of consolation among 3680 dyads. Quadratic assignment procedure.
(DOCX)

S7 Table Logit analysis of consolation among 3390 dyads. Quadratic assignment procedure.
(DOCX)
