# Supplementary material for: Consolation in the aftermath of robberies resembles post-aggression consolation in chimpanzees
Source: PLoS One. 2017 May 31;12(5):e0177725. doi: 10.1371/journal.pone.0177725 (PMC5451014; doi:10.1371/journal.pone.0177725)
Supplement: S1 Table — (DOCX) [file pone.0177725.s003.docx]

S1 Table: Codesheet for static variables during aftermath of robbery

| Variable | Categories | Instructions |
| --- | --- | --- |
| Location | Supermarket, Night store or Minimart, Bar or Restaurant, Hotel, Jewelry store, Other | Based on the appearance of the setting. |
| Location size | Small, Large, Unknown | Small if would take an average person less than 5 seconds to walk across the room, large if it would take longer. Unknown if it cannot be established |
| Gender | Male, Female, Unknown | Base judgment on body form, hair, clothing and other body wear |
| Role | Employee, Customer, Unknown | Base judgment based on clothing, position in the room (e.g. behind desk) or on the type of activity the person displayed |
| Age | In years | Estimate on body shape, speed of movement, baldness (males) |
| Ethnicity | White, Black, Moroccan/Turk, Asian, Hindustan, Other, Unknown | Best guess based on skin color, hair color, hair style, complexion and clothing. |
| Degree of threat | Weapon threat and physical force  Physical force, Weapon threat, Within reach of offender, None  Unknown | Categories are ordered from most serious to least serious. Base judgment on most serious victimization during the whole robbery event. Physical force includes use of any weapon, kick, hit, grab or push aggressively |
