# Supplementary material for: Consolation in the aftermath of robberies resembles post-aggression consolation in chimpanzees
Source: PLoS One. 2017 May 31;12(5):e0177725. doi: 10.1371/journal.pone.0177725 (PMC5451014; doi:10.1371/journal.pone.0177725)
Supplement: S2 Table — Partial ethogram of offender behavior. (DOCX) [file pone.0177725.s004.docx]

S2 Table: Codesheet of dynamic variables during robbery. Partial ethogram of offender behavior.

| Action | Towards |  |
| --- | --- | --- |
| Physical force | Target of force | power, violence, or pressure directed against an individual consisting in a physical act power, violence, or pressure directed against an individual consisting in a physical act including kick, hit, throw, push or grab aggressively, including with a weapon |
| Weapon threat | Target of threat | an object that can inflict bodily harm is pointed at someone |
| Threat and force | Target | both physical force and weapon threat |
| Within reach | Target (person within 2 meter of offender) | was close enough to offender(s) to be touched (within arm’s reach; approximately 2 meters, without actual threat or force) |
