# Supplementary material for: Consolation in the aftermath of robberies resembles post-aggression consolation in chimpanzees
Source: PLoS One. 2017 May 31;12(5):e0177725. doi: 10.1371/journal.pone.0177725 (PMC5451014; doi:10.1371/journal.pone.0177725)
Supplement: S3 Table — (DOCX) [file pone.0177725.s005.docx]

S3 Table: Codesheet for dynamic variables during aftermath of robbery (ethogram)

| Action | Towards | Description |
| --- | --- | --- |
| Present at scene |  | Person enters CCTV-footage. Used at one point during observation |
| Light consolation | Target of consolation | Touch target’s arms, hands |
| Strong consolation | Target of consolation | Touch target’s upper body, including head |
