# Supplementary material for: Consolation in the aftermath of robberies resembles post-aggression consolation in chimpanzees
Source: PLoS One. 2017 May 31;12(5):e0177725. doi: 10.1371/journal.pone.0177725 (PMC5451014; doi:10.1371/journal.pone.0177725)
Supplement: S4 Table — Quadratic assignment procedure. (DOCX) [file pone.0177725.s006.docx]

S4 Table: Logit analysis of consolation among 3680 dyads. Quadratic assignment procedure.

| Explanatory variable | b^1^ | or^2^ | p^3^ |
| --- | --- | --- | --- |
| Socially close | 1.922 | 6.83 | .000 |
| Potential provider female | .958 | 2.61 | .002 |
| Potential recipient is victim | 3.561 | 35.20 | .000 |
| Number of subjects in aftermath | .021 | 1.02 | .000 |
| Physically close ( < 2m) | .330 | 1.39 | n.s. |

^1^ estimate (average of 1000 iterations)

^2^ odds ratio = exp(b)

^3^ p-value (based on 1000 iterations), n.s. = p ≥ .050
