# Supplementary material for: Consolation in the aftermath of robberies resembles post-aggression consolation in chimpanzees
Source: PLoS One. 2017 May 31;12(5):e0177725. doi: 10.1371/journal.pone.0177725 (PMC5451014; doi:10.1371/journal.pone.0177725)
Supplement: S6 Table — Quadratic assignment procedure. (DOCX) [file pone.0177725.s008.docx]

S6 Table: Logit analysis of consolation among 3680 dyads. Quadratic assignment procedure.

| Explanatory variable | b^1^ | or^2^ | p^3^ |
| --- | --- | --- | --- |
| Socially close | 2.009 | 7,46 | .000 |
| Potential provider female | .954 | 2,60 | .001 |
| Potential recipient is victim | 3.634 | 37,86 | .000 |
| Number of subjects in aftermath | .036 | 1,04 | .000 |
| Potential recipient female | .560 | 1,75 | n.s. |

^1^ estimate (average of 1000 iterations)

^2^ odds ratio = exp(b)

^3^ p-value (based on 1000 iterations), n.s. = p ≥ .050
